# Investigation on the acquisition of scientific competences during medical studies and the medical doctoral thesis

**DOI:** 10.3205/zma001167

**Published:** 2018-05-15

**Authors:** Nurith Epstein, Johanna Huber, Martin Gartmeier, Pascal O. Berberat, Maike Reimer, Martin R. Fischer

**Affiliations:** 1 LMU Munich, University Hospital, Institute for Medical Education, Munich, Germany; 2 Technical University of Munich, University Hospital Rechts der Isar, TUM Medical Education Center, Munich, Germany; 3 Bavarian State Institute for Higher Education Research and Planning (IHF), Munich, Germany

**Keywords:** research competences, medical doctoral thesis, medical curriculum

## Abstract

**Background: **Not only the amount of research related contents in German medical studies are objects of criticism, but also the medical doctoral thesis. However, the question which research competences are truly acquired within medical school and the doctoral phase is empirically open, and is thus pursued in the following research study.

**Methods: **We used data from the Bavarian Medical Graduate Panel Survey (MediBAP) (N=455), where respondents assessed their own research competences. To consolidate the data, we analysed qualitative interviews with doctoral medical graduates and students from the E-Prom study (N=14).

**Results: **The quantitative analyses show that medical graduates evaluate the medical curriculum's research contents and their acquired competences as rather low. Doctoral graduates rate their competence of pursuing research independently higher than medical graduates who have not finished their doctorate. The qualitative analyses are in line with these results, as they point to the predominant development of competences during the doctoral phase. Despite this clearly positive trend, the majority of the respondents don't feel confident enough to undertake research independently also after attaining their doctoral degree.

**Conclusion:** The results of this study emphasize the need for a more systematic and targeted mediation and review of research competences within the regular medical curriculum.

## Zusammenfassung

**Hintergrund: **Das Medizinstudium in Deutschland steht in Bezug auf seine wissenschaftlichen Inhalte ebenso in der Kritik wie die medizinische Promotion. Welche wissenschaftlichen Kompetenzen Studierende der Medizin im Rahmen des Studiums und der Promotion tatsächlich erlernen ist jedoch eine empirisch offene Frage, der die folgende Arbeit nachgehen möchte.

**Methoden:** Zunächst wurden Daten des bayerischen Mediziner/innen-Absolventenpanels (MediBAP) herangezogen (N=455), in dem Befragte ihre Kompetenzen in Bezug auf übergreifende wissenschaftsbezogene Kompetenzen einschätzten. Zur Vertiefung der Daten wurden qualitative Interviews der E-Prom Studie mit promovierten und promovierenden Medizinerinnen und Medizinern ausgewertet (N=14).

**Ergebnisse: **Die quantitativen Auswertungen weisen auf eine geringe Bewertung wissenschaftlicher Studieninhalte sowie eine generell geringe wissenschaftliche Kompetenzeinschätzung hin. Promovierte schätzen ihre Kompetenzen in den Bereichen des eigenständigen Forschens signifikant besser ein. Im Einklang mit diesen Ergebnissen stehen die qualitativen Analysen, die auf die vorwiegende Kompetenzentwicklung in der Promotionsphase hindeuten. Trotz der deutlich positiven Entwicklung im Rahmen der Promotion traut sich der Großteil der Befragten am Ende der Promotionsphase keine selbständige Forschungstätigkeit zu.

**Schlussfolgerung:** Die Ergebnisse dieser Arbeit unterstreichen, dass eine systematischere und zielorientiertere Vermittlung und Überprüfung wissenschaftlicher Kompetenzen im Medizinstudium dringend notwendig sind.

## 1. Background

Inadequate imparting of research competences during medical studies is the object of repeated complaints from experts and medical specialists. Indeed, the Deutsche Forschungsgemeinschaft (German Research Foundation) [1] draws attention to the fact that,* “if an undergraduate university course imparts professional skills, but does not also provide basic scientific/research training, ... the organisation of this course must be reconsidered”* (ibid. p. 3). The Wissenschaftsrat (Scientific Council) also recently criticised the lack of research contents in medical studies [2]. This criticism was directed towards model/reformed medical studies, where only a few positive measures in the direction of science/research were identified. It should also be noted that medical students don’t expect to learn much in the area of research: only one third of them expects their studies to *“enable them to independently apply scientific research methods”* ([3], p.138). 

Considering this problem, it appears paradoxical that the majority of medical students attain a doctoral degree: the percentage of medical graduates with a doctoral title varies between 60 [4] und 80% [5], [6]. However, the doctoral thesis in medicine is usually started during the regular medical curriculum [7], and is often finished right after graduation. Critics therefore raise the question of whether it is even possible to write a doctoral thesis that complies with the qualitative standards of other neighbouring disciplines, while being simultaneously a regular student in medical school [5]. Furthermore, a dissertation's quality should accurately mirror the research competences that were acquired by the doctoral student/graduate. The well-known methodical diversity of medical dissertations (e.g., experimental, clinical, statistical, theoretical) does not guarantee any specific conclusion on the quality or level of aspiration of a thesis. While several individual medical doctoral theses with questionable contents and of questionable quality are being discussed in the press (e.g. [8]), there is still no systematic, empirical review of the quality of medical doctoral theses. Although, in some faculties at least, the proportion of medical dissertations published in the form of journal articles has increased during the past few years [9], [10], [11], their publication was recently provocatively referred to as “a contamination of research literature” („Kontamination der Forschungsliteratur“) [12]. 

There are also a few smaller, local research studies, where doctoral medical students are asked about the meaningfulness of their doctoral thesis [9], [10], [13]. A large proportion of the interviewees state that the doctoral thesis would indeed have been meaningful, but that does not allow us to deduce which competences were actually acquired. Meaningfulness could also refer to individually perceived career opportunities, or the acquisition of competences unrelated to research. As part of another research study with medical doctoral students from five faculties in Baden-Württemberg, the majority of respondents stated that they had pursued a doctorate, merely because it is common practice in medicine [14].

In this context, a comparative study in which, medical doctoral graduates and doctoral graduates from other life sciences were interviewed, has meaningful results. Shortly after attaining their doctoral degree, medical doctoral graduates had significantly lower publication productivity and indicated a lower perceived research self-efficacy [15], meaning they felt less confident about carrying out successfully research-related activities such as publishing or applying for third-party funding. Because doctoral graduates were interviewed shortly after graduation, one can assume that their different experiences during medical studies and the doctorate led to a worse result. However, it is possible that also first occupational experiences are reflected in this self-assessment. 

In accordance with the criticism about medical studies’ research related contents, and the evidence of a less pronounced research interest among medical students [15], [16], there is also the complaint of a lack of physician scientists (cf. summarised in [13]). However, acquiring research competences is crucial for all medical students, not only in order to pursue research careers, but also to practice evidence-based medicine: integrating the best scientific evidence, clinical expertise, patient values and wishes [2]. The point is not to train all physicians to become “top scientists” (“Hochleistungswissenschaftlern”) [17], but to implement a solid foundation of research competences, in addition to clinical and social skills. It remains questionable as to what extent research competences are taught and learnt sufficiently during medical studies and the doctorate, so that on the one hand, graduates are able to carry out their clinical activities evidence-based; and on the other hand, to pursue research activities within their clinical careers or even focus on an academic research career. Thus, this article explores the following question: which research competences are being imparted or acquired during medical studies and the doctorate? Since research competences are considered very important for physicians, but the latter's lack of interest in research is an object of criticism, the article seeks to also reveal which factors influence the acquisition of research competences. 

## 2. Methods

In order to answer this question, data from two different sources was drawn, the first data source being quantitative data from the Bavarian medical graduate panel (Bayerisches Absolventenpanel der Medizin – MediBAP). Here, participants evaluated the scientific content of their studies and their own scientific skills. Within the qualitative interviews of the E-Prom study [http://www.klinikum.uni-muenchen.de/E-Prom/de/index.html] medical doctoral graduates and medical doctoral students were interviewed (cf. [18]).The qualitative data completes the MediBAP data, regarding the evaluation of attaining research competences during the course of medical studies and the doctorate. Since participants were asked about their development of research competences within the regular medical curriculum and the doctoral phase, the data allow us to better differentiate between those contexts. Furthermore, the data allow for a detailed determination of acquired and non-acquired research competences, being construed to accept free answers. The methods and results of both studies will be presented and are followed up by an integrative discussion of the results.

### 2.1. Quantitative study (MediBAP)

#### 2.1.1. Sample

The MediBAP was conducted for the first time in the winter of 2015/16 by the Bayerisches Staatsinstitut für Hochschulforschung und Hochschulplanung (IHF) (Bavarian State Institute of University Research and Planning), in cooperation with the research group for quality management and graduates' survey (Q & A), belonging to the Kompetenznetz Medizinlehre Bayern (KMB) (Bavarian Competence Network for Medical Education) [19]. In the context of the MediBAP, graduates who had completed their human, dentistry or veterinary medicine studies at one of the five Bavarian Faculties of medicine (FAU Erlangen-Nürnberg, LMU Munich, TU Munich, University of Regensburg or JMU Würzburg) between the 1rst of October 2014 and the 30th of September 2015 (cf. ibid. [1]) were interviewed. The survey was conducted during the 2015/16 winter term [19]. To answer the central question of this research paper, we used the survey data of graduates from human medicine (N=479). The gender distribution (65% female subjects) corresponds well to the nationwide average in the field of medicine [20]. The average length of studies in the sample (13.2 semesters) is slightly higher than the overall average [11]. [8]; however there are no differences in the grade point average (GPA) ([18], p. 8). In the sample, 69% of respondents are currently working on their doctorate, 21% have already completed it, 4% are still planning it and 3% state that they have no intention to pursue one at all. This overall situation is comparable to the data in other research studies (see Chapter 1).

##### 2.1.2. Operationalisation and data analysis 

The MediBAP study included two sets of questions concerning the acquisition of research competences [21]. First of all, the participants had to assess two aspects of the teaching of research competences during their studies (the training of research methods and writing of scientific texts). Subsequently, the scale “Competences to act evidence-based” (“Kompetenzen zum wissenschaftlichen Handeln”) from the Freiburger Kompetenzfragebogen (Freiburg Competence Questionnaire) was used in order to find out to what extent different research competences were acquired during the regular medical curriculum [22]. The assessment of the items was carried out on a five-point Likert scale, with higher values corresponding to a higher level of agreement (5=to a high extent, 1=to a low extent). Another single item in the questionnaire referred to the extent to which medical studies prepare to act and think evidence-based in their current occupational position. Since all these items corresponded to different research competences and evaluations of the medical curriculum, they were considered individually and were not summarized into scales. 

The items referring to the medical curriculum were analysed for the total sample, the acquired research competences and the ability to act evidence-based in the professional world were analysed by doctoral status(doctoral graduates vs. no doctoral graduates). 

For analyses performed by doctoral status, the group of doctoral students was not considered, as it was suspected that there was great heterogeneity in the progress of their doctorates. Differences between doctoral and non-doctoral students are tested for significance with the help of t-tests. Individuals who did not state their doctoral status (N=24) were not included in the analyses. The number of cases was kept constant in the questionnaires, so that only individuals who didn't have any missing values in the items of a questionnaire were considered.

#### 2.2. Qualitative study (e-prom)

##### 2.2.1. Sample

In the context of the E-Prom study, ten doctoral graduates and four doctoral students were interviewed (cf. also [13], [15]). The interviews were conducted between October 2014 and November 2015. Table 1 (Tab. 1) conveys an overview of the study participants.

2.2.2 Instruments of Data Collection and Analysis 

The interviews were conducted with the help of structured interview guidelines. The guideline was structured according to the interviewees' academic careers, starting with their admission into medical school, and finishing with the start of their professional career and their career goals (cf. [13], [15]). The interviews were transcribed verbatim. A coding scheme was developed mainly deductively (cf. [13], [15] and attachment 1 ), following Mayring's method of qualitative content analysis [23], some subcategories were formed inductively during the coding. The coding was done sentence by sentence. Moreover, the whole interview was coded for all the categories, as the interviewees did not only make relevant statements after a specific question was asked. Based on these codings performed in the context of the E-Prom study, only the passages which referred directly to the acquisition of research competences within both the medical study and the doctoral phases were analysed and interpreted. The most relevant categories were: “the acquisition of research competences during medical studies”, “the lack of research competences after and during the doctoral phase”, “reasons for the lack of research competences”, “new competences after the doctoral thesis” and “the relevance of acquired competences for the future career”. The interrater reliability of two independent raters was calculated using Cohen's kappa [23] (cf. [13], [15], and, corresponding to the categories described in the appendix, had a value of 0.86 [24], [25], thus considered “good”. The calculation of Cohen's kappa was based on the presence of the code as a measure of agreement, since the exact location in the interview was irrelevant to the interpretation. The categories and their associated subcategories are listed in the attachment 1 . 

## 3. Results

### 3.1. Quantitative results

#### Research related content within the medical curriculum

The results of the evaluation of research related contents in the regular medical curriculum are illustrated in Table 2 (Tab. 2). Overall, medical graduates rate the training of research methods within medical school slightly below the scale midpoint of three. Learning how to write scientific texts indicates an even lower overall result. These values related to the training of research competences have a significantly lower rating than values recorded for the acquisition of clinical skills in medical school [20]. As an example, the mean for the acquisition of “general skills, competences and abilities related to differential diagnostic thinking” is 3.5. 

The rating of medical school with respect to preparing students to act evidence based within their professional life is depicted in Table 3 (Tab. 3). In the total sample, as well as in all subgroups, the item is rated slightly below the scale midpoint. Thus, just like other items relating to the acquisition of research competences, being prepared for evidence-based clinical practice, has been assigned a lower value, than the items corresponding to the acquisition of clinical skills. 

##### Evaluation of own research competences 

The items related to research competences from the Freiburger Kompetenz-Fragebogen (Freiburg competence questionnaire), are represented in Table 4 (Tab. 4). The ability to question one's own and others' ideas, and the ability to understand and classify medical information from the lay press, have a much higher overall value in comparison to the other items on the scale. This could be due to the fact that these items do not reflect concrete aspects of performing research as the previous two items on the scale (such designing a study, generating hypotheses, and choosing methods of analyses). The decision to participate in a study based on methodical and ethical aspects, and the ability to do research independently, get lower scores. The only significant differences between medical graduates who have and those who have not attained their doctorate, is in their assessment of their ability to perform research independently (*t(129)=3.74; p=0.000*), with medical doctoral graduates stating that they feel more confident about it.

#### 3.2. Qualitative results

##### Acquisition of research competences during medical school

Even though the study participants reported that some research competences were imparted to them during their studies, e.g. during classes on statistics and epidemiology, or during work placements in laboratories, the overall increase of competence was rated as low. The respondents associated research competences with independent work and thought. They saw this aspect as under-represented due to the very regimented study plan:

Right... We didn't really learn that kind of … scientific work, I have to say. It was like school – here is the book, this is what you have to learn – learn it, know it, answer the questions – and that's it. (Interview 1415, Sections 32-34). 

When we asked about the acquisition of research competences during medical studies, it became clear that for most participants, this process really took off only when they started working on their doctoral thesis:

Well for me, it was like, sure, we occasionally get to know a clinical study or a study result , but actually doing research only really took place during my doctoral thesis. (Interview 8, Section 37). 

##### The acquisition of research competences during the doctoral phase

Furthermore, the respondents were interviewed on their acquisition of research skills during the doctoral phase. The interview focused on two aspects: the acquisition of new competences and the lack of certain competences. In that respect, the participants were asked which competences they lacked *during* and *then after the completion of their doctoral thesis*. The first aspect is related to the regular medical curriculum, the second aspect is relevant for the evidence-based practice of medicine as well as academic research careers. First of all, we will discuss the aspect of lacking research competences during the doctoral phase; then, we will present the acquired skills during that phase, and finally, the lacking research competences after attaining the doctorate. 

##### Lacking research competences during the doctoral phase

Participants specifically mentioned problems with scientific writing. These problems were often directly linked to missing general knowledge of the field of research, which was dealt with in the dissertation. Moreover, several difficulties have been reported, which had mainly to do with writing the “discussion” section, for which a solid overview of the research topic, the evaluation and classification of the results, are crucial. 

For me, writing the introduction, presenting the material and methods was very easy. The result analysis and discussion was much harder to write, contentwise. (Interview 22, section 155).

[…] But I think a medical student can't write, for example, the discussion part properly in the beginning, that is, in a way that other scientists would accept it as legitimate. (Interview 21, section 178-180). 

Participants also felt uncomfortable about language and style itself that is, writing scientifically, not only in German, but also in English. They reported difficulties in presenting their results on paper, since they didn't really have to write scientific texts before beginning their doctoral thesis, during their studies. 

How do I write this correctly or phrase it well? – that was pretty difficult, especially since it had to be in English […] and then phrasing it scientifically and not writing things that are only half true and sound like a child in kindergarten wrote them. (Interview 21, sections 187 and 190). 

##### New acquired competences after the doctorate

It should be noted that overall, the acquisition of competences in the context of writing the doctoral thesis very strongly depends on personal motives and research interest in the first place. These, in turn, influence the choice of the topic, so that high motivation in learning leads to the choice of a complex thesis topic or, and the chosen topic is also worked on with a higher ambition. One interviewee, who was not so interested in research in the first place, admits that her research competences evolved on a rather low level, and that some have already been forgotten.

When asked about specific new skills they acquired, participants mentioned methodology (how to design a research study) and methods (e.g. statistical data analysis and laboratory techniques).

Methodological aspects, especially setting up a lab experiment, are important not only for one's own research, but also for understanding and classifying research literature and proper results in the overarching context. Moreover, participants also talk about skills related to publishing research. This includes scientific writing, with all the formal aspects (formatting, quoting sources correctly), but also how to deal with reviewer comments. While participants report a gain in competences in this area, it became clear that our interviewees did not feel ready to conduct research or publish completely on their own, even after completing their doctoral thesis.

I'd say, competent, well I'd say it could always be better. I feel competent enough to submit a very acceptable paper to my boss. But we'd have to discuss it then. (Interview 8, sections 145-147).

Participants stated that they acquired some skills whose importance goes beyond doing research. They mentioned to have gained a “scientific mindset” i.e., a systematic work approach to solving problems, a higher ability to cope with failure, autonomous work and a sense of responsibility. As mentioned earlier, their own motivation was seen by the participants as an important factor for growing their competences. Furthermore, the support of the supervising professor and other people also plays a crucial role. 

I'd say that my personal interest and ambition definitely played a role in it. Looking at my fellow students who are taking forever in writing their thesis, I'd say that research and, more generally, learning to use research methods, has a lot to do with personal interest and the willpower to do it, to be able to do it. I'd also include my supervising professor here, as he was supportive and showed me the right way, and my parents, who gave me a lot of advice while I was writing it. (Interview 1, section 110).

When looking at one's own motivation, it becomes apparent how important independent work is, and how it presupposes a certain degree of a self-efficacy, meaning to have confidence in one’s abilities to learn new things and to apply them. 

I was really doing something – myself. Sitting down by myself, working with the data, the statistics program and finding out what kind of results I get, evaluating those results. […] I think it was really important that I did something here all on my own. (Interview 11, section 118).

##### Missing competences after the doctorate 

Very frequently, methodical competences, independent scientific writing and the composition of scientific journal articles in particular – including articles in English, – presenting scientific results (e.g. during conferences), communicating with other scientists, or the supervision of doctoral candidates are brought up in this regard.

Participants see the narrow focus of the doctoral thesis's topic as a major cause for their lack of methodical competence. For example, a doctoral student working on a clinical questionnaire research study does not know how to do research involving animal experiments. Participants pointed to shortcomings in the methods applied in their own thesis as well. They said that their knowledge certainly increased, in the area of statistics for instance, but they also pointed out that they were still far from being experts in the field. Again, this was seen in connection with the narrow research question of their doctoral thesis: 

I think that when it comes to evaluating statistics, I really only learned the basics that I needed for my work, and that is sort of the problem. I can't say that I have any sort of comprehensive knowledge in the field. If I really wanted to go for an academic research career, I'd definitely have to do a lot more in statistics. (Interview 22, sections 144-146).

The main causes for some missing skills are attributed to the narrowness of doctoral thesis' topics, and also to the fact that most interviewees spend relatively little time in their scientific communities. Applying for research funding, working with cooperation partners, or supervising final papers are other competences that are affected by these factors. Our study participants explain the lack of these competences by the structure of the medical doctoral phase. Furthermore, within the field of medicine, these competences mostly become relevant only after the completion of the thesis. That's why interviewees who have already completed their thesis but were still active in research mostly mentioned a lack of experience in these areas. 

Overall it can be said that the acquisition, or the lack of competences, largely depend, as expected, on the candidate's personal initiative and ambition; candidates with a higher research interest tend to go into areas that already relate to an academic research career – they also work in greater detail and on a higher level with skills or future challenges.

##### Judging the relevance of research competences 

The majority of respondents’ views the collected experience and attained competences within the doctoral phase as being significant and relevant to their future career. This opinion is even stronger (as would be expected) in medical professionals still active in research; the knowledge they gained is a prerequisite for any further research they carry out. Participants who have not pursued research further rather emphasize general competences attained during the doctorate, such as working independently, analytical thinking, having a sense of responsibility, and a better understanding of people around them.

Yeah, definitely. You have to work independently in the clinic as well and of course, you need to think and act critically. And taking responsibility, being conscious of that, definitely. (Interview 18, section 167).

The significance of these acquired research competences for practicing evidence-based medicine is not addressed as frequently by the participants. 

[…] If I want to participate in what is generally happening, then I definitely have to stay on top of

the newest research which can't just be found in a textbook. […] I think that's something that really helped me. (Interview 2, section 79). 

## 4. Discussion

The MediBAP data analysis and, in particular, the analysis of the qualitative interviews with medical doctoral graduates , indicate that the acquisition of research competences in medical school is only weakly established, and takes place predominantly in the doctoral phase. Since, unlike in other disciplines, the doctoral thesis is the first written scientific work, correct scientific work methods remain yet to be learnt. These include formalities such as formatting, correct citation, but also building up a scientific research paper, and the use of appropriate scientific terminology and writing style. Scientific writing, an essential scientific skill, is particularly difficult for medical doctoral students. This is clearly shown in the quantitative data: here, “learning how to write scientific texts” during medical studies, and “practising research methods” are rated as low. Thus, the presence of these problems during the doctoral phase is not surprising.

In many cases, problems arise when writing specific parts of the paper/thesis which require a classification into the research literature (interpretation and discussion of results). This indicates that it is challenging for the doctoral candidates to obtain a comprehensive research overview of their topic within the context of their doctoral thesis. This result is yet another proof of inadequate teaching of basic research competences in medical studies, which is also reported by the respondents. It would make sense to compare this result with those of other life science subjects, in order to be able to classify it better. Since studying other life sciences disciplines requires students to write other scientific essays (Bachelor and Master theses but also seminar essays etc.) before graduation and before starting a PhD, the assessment of these competences is likely to be higher. 

The analysis of the qualitative data reveals, however, that medical students do acquire a variety of scientific competences during their doctoral phase. In this case, doctoral students mention in particular methodical competences and methodology, which then allow them to evaluate research studies. Assessing clinical studies is a central aspect of evidence-based practice. The acquisition of this competence is very clearly attributed to the doctoral phase. However, physicians who have not attained a doctoral degree should master this skill as well. According to the MediBAP data, on average, research related competences are rated lower than the scale midpoint. Also compared to the assessment of other clinical competences, one can consider these ratings to be rather low (cf. [16]). In the qualitative interviews, respondents with a lower research interest see the usefulness and value of the doctoral thesis for clinical activity with respect to having learnt to, i.e. work independently, having attained social and communicative “soft skills”, such as dealing with patients. The relevance of assessing new research study results for their clinical activities is rarely mentioned. Further studies should examine the extent to which medical students and practicing physicians are aware of the concept of evidence-based medicine.

Although the graduates having acquired their doctorate report an increase in competence in areas, such as working independently, knowing the structure of a scientific paper, quoting correctly etc., some knowledge deficits are present nonetheless. The participants refer in particular to difficulties in classifying/evaluating their research results on their own, and in association with this, mention trouble in writing certain aspects of their doctoral thesis. In addition, the development of research competences is focused on the specific research question of their doctoral project. By consequence, the medical doctoral thesis cannot, by its nature, provide broad, general research training. 

Another important aspect is that the increase in research competences is strongly related to the doctoral students' individual motivation and research interest, and therefore varies greatly between the respondents. Thus, the doctorate does not enable all doctoral students to work independently, and cannot fulfil its function in all of the cases. In this context, it is also clear how crucial it is to intensify the “efforts of many medical faculties to improve the quality of medical doctoral theses” [7]. For example, the Deutsche Hochschulverband (German Association of Universities) proposes to give out only “topics for medical dissertations, which make a substantial contribution to the progress of scientific knowledge.” (cf. Ibid). Such a measure would at least reduce the possibility of writing a “thesis which is very limited in scope”. 

In accordance with the qualitative results, the MediBAP data include the assessment of independent research competences, in comparison to more generalized scientific competences, such as i.e. classifying medical information from the lay press. This assessment was rated as rather low, even for medical students having already attained their doctor's title, nonetheless (as expected), it was still much higher than for medical students without a doctorate. 

The item formulations result in a limitation of the quantitative data results' validity. Future studies about physicians' research competences should consider establishing items that capture more concrete research competences. For example, dealing with medical information from the lay press is an important indicator of evidence-based clinical practice, but in terms of research competence, understanding and classifying results from primary research is particularly important. Other items addressing research competences could also be rendered more precise and exact. Thus, the concept of practising research methods is formulated very vaguely, as research consists of many different aspects and stages. In the future, it would be preferable to be able to differentiate such aspects, as well as the acquisition of research competences within the regular medical curriculum and the doctoral phase. Because of the diversity of research competences, it would be helpful to understand and define which competences should be learnt in different phases of medical studies. The Nationale Kompetenzbasierte Lernzielkatalog Medizin (NKLM) (National Competence-Based Learning Objectives Catalogue for Undergraduate Medical Education) already includes a detailed list of “medical-scientific/research related competences”, which can serve as a basis for future research studies on the topic [http://www.nklm.de]. 

In order to evaluate comprehensively and differentially the quality of medical doctoral theses, direct content-based analyses of medical dissertations would be required. In conclusion, one can state that this research study also supports the much-discussed lack of research competences among future physicians. This emphasizes the need to teach and assess research competences in medicine, in a systematic and goal-oriented way. In addition, the question arises, as to how medical doctoral theses will be structured and carried out in medical faculties, in the future. This includes the selection of applicants and “transparent procedures for quality assurance” [7]. 

## Notes

The survey of Bavarian medical graduates, the Bayerische Absolventenpanel Medizin (MediBAP) was carried out in the context of the working group Qualitätsmanagement und Absolventenbefragungen of the Kompetenznetz Medizinlehre Bayern, funded by the Bayerische Staatsministerium für Bildung und Kultus, Wissenschaft und Kunst, in cooperation with the Bayerisches Staatsinstitut für Hochschulforschung und Hochschulplanung (IHF).

The E-Prom study was funded by the Bundesministerium für Bildung und Forschung (BMBF). 

## Acknowledgements

We thank all responsible people and partners of the E-Prom and MediBAP projects, for their friendly cooperation and the transfer of the research data. In this context we would like also to thank the participants of both studies.

## Competing interests

The authors declare that they have no competing interests. 

## Supplementary Material

Overview of the Coding Scheme

## Figures and Tables

**Table 1:**
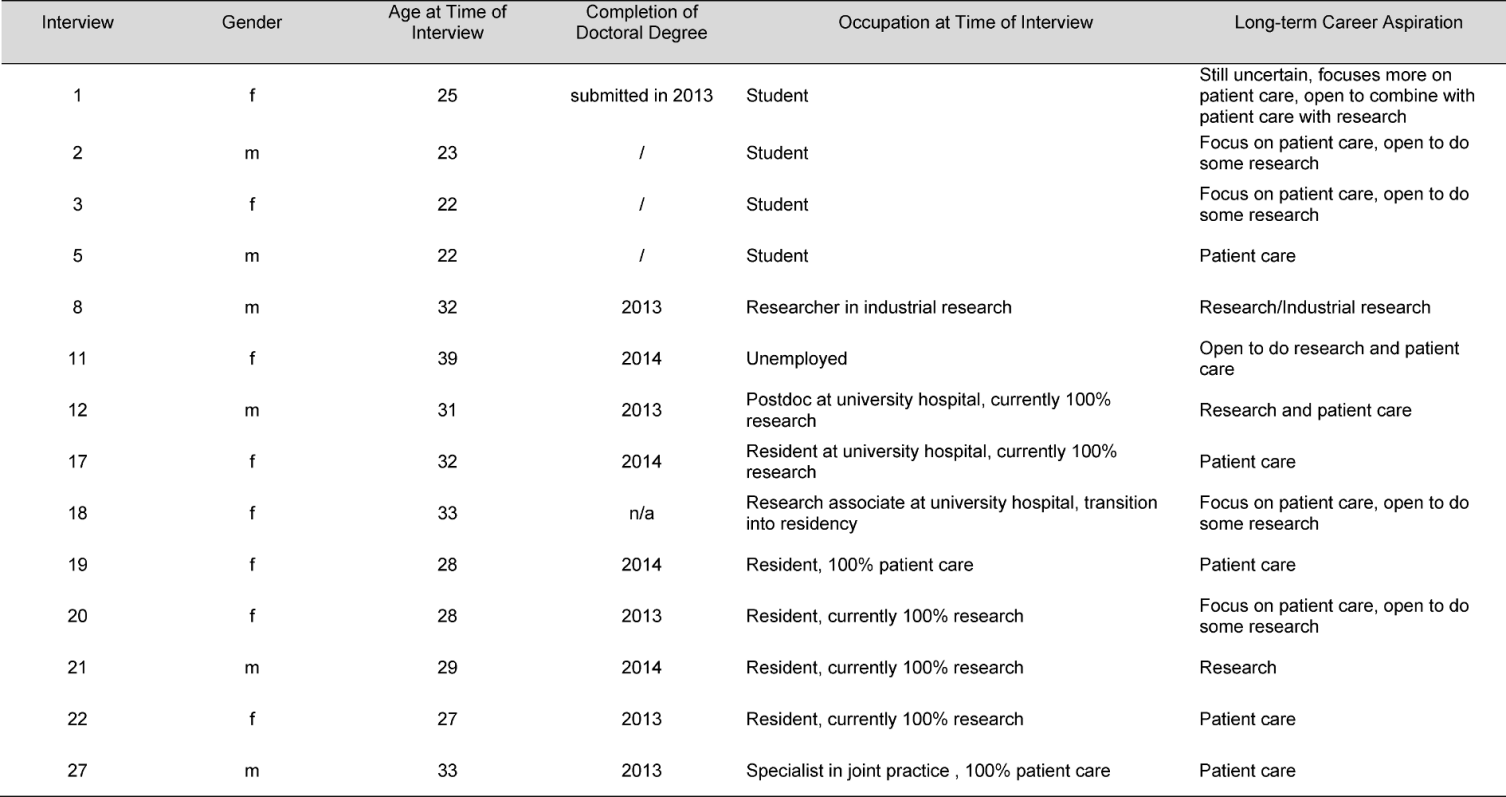
Overview of the E-Prom study participants

**Table 2:**
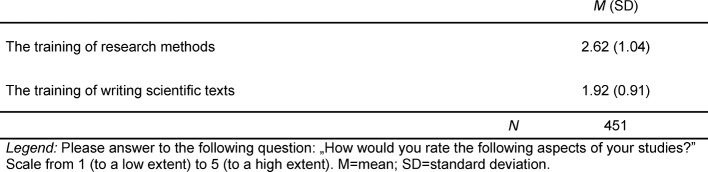
Assessment of Research Training within Medical Studies

**Table 3:**
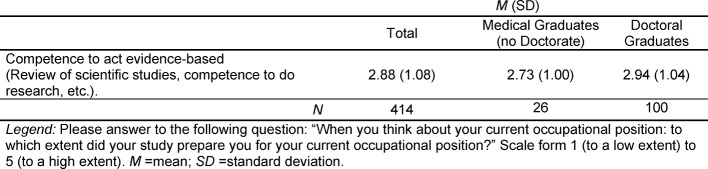
Medical Studies as Preparation to Act Evidence-Based in the Current Occupational Position

**Table 4:**
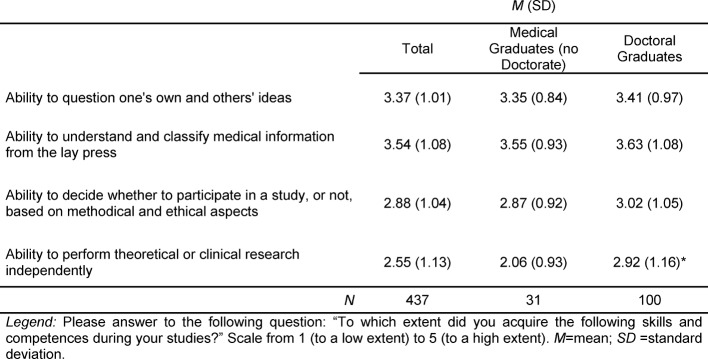
Freiburger Kompetenzfragebogen, Assessment of Research Competences
