# Supplementary material for: Investigation on the acquisition of scientific competences during medical studies and the medical doctoral thesis
Source: GMS J Med Educ. 2018 May 15;35(2):Doc20. doi: 10.3205/zma001167 (PMC6022582; doi:10.3205/zma001167)
Supplement: Overview of the Coding Scheme [file JME-35-20-s-001.pdf]

Main Categories	Subcategories
Acquisition of research competences during medical studies	low
	medium
	high
Lack of research competences after and during the doctoral phase	clinical experience
	knowledge of research literature
	methodical competence
	ability to manage and plan a research project
	scientific writing
	scientific English
	presenting research results
	writing grant proposals
	supervision of doctoral candidates
	social skills
	other
Reasons for the lack of competences	unrealistic at that time
	context of the doctoral project
	no opportunities
	own abilities
	learning opportunities were not taken despite being interested
	learning opportunities were not taken because of a lack of interest
	other
New competences after the doctorate	general increase in competences clinical experience
	knowledge of research literature
	methodical competence
	project planning
	scientific writing

	scientific English<br>publication process<br>presenting of research results<br>writing grant proposals<br>scientific thinking and reasoning<br>social skills<br>other
Which factors were important for the development of research competences	encouragement from the supervisor<br>encouragement from other people<br>exchange of thoughts and ideas with other scientists<br>own motivation<br>amount of own efforts<br>seminars/courses<br>other
Relevance of acquired competences for (future) career	rather irrelevant<br>rather relevant<br>highly relevant
